# Low envelope fluctuation OFDM waveform design under subcarrier constraints

**DOI:** 10.1038/s41598-026-48619-8

**Published:** 2026-04-16

**Authors:** Mingxing Fu, Defu Jiang, Kanghui Jiang, Yiyue Gao, Yan Han

**Affiliations:** 1 School of Information Science and Engineering, Hohai University, Nanjing, 210098 China; 2 Laboratory of Array and Information Processing, Hohai University, Nanjing, 210098 China; 3 School of Electrical and Power Engineering, Hohai University, Nanjing, 210098 China

**Keywords:** OFDM waveform design, envelope fluctuation suppression, coefficient of variance of envelope (CVE), subcarrier-related constraints, Engineering, Electrical and electronic engineering

## Abstract

In this paper, we propose an orthogonal frequency-division multiplexing (OFDM) weight design mechanism aimed at reducing envelope fluctuations under subcarrier constraints. In the time domain, a new metric is introduced and minimized to guide the OFDM waveform toward a constant-envelope structure. In the frequency domain, the design allows a subset of subcarrier weights to be pre-assigned and imposes upper bounds on the magnitudes of the remaining subcarrier weights. Specifically, we formulate and solve an optimization problem that minimizes the variance of the envelope distribution, subject to subcarrier constraints and a fixed total energy budget. This leads to a non-convex optimization problem. By relaxing certain constraints−specifically, the upper-bound constraints on weight magnitudes−and subsequently reintroducing them, we transform the original non-convex problem into an unconstrained maximization over a unimodular complex vector and develop an iterative solution approach that converges to a local optimum. In addition, the effect of the initial point selection on the resulting weights is analyzed, which motivates a heuristic strategy for roughly controlling weight magnitudes. Numerical results demonstrate that the proposed method effectively suppresses envelope fluctuations while satisfying subcarrier constraints. Compared to existing methods, the proposed mechanism achieves higher computational efficiency and a better approximation to a constant-envelope signal.

## Introduction

In radar systems, multicarrier signals implemented using orthogonal frequency-division multiplexing (OFDM) have been extensively investigated^1–5^ due to their flexible spectrum shaping capability^6,7^ and enhanced target detection enabled by frequency diversity^8^. However, OFDM signals inherently exhibit large envelope fluctuations, which are undesirable for radar transmitters. In practical radar front-ends, RF power amplifiers typically operate close to saturation to achieve high efficiency^9^. Fluctuating input envelopes therefore induce nonlinear distortions, including amplitude modulation–to–amplitude modulation (AM/AM) and amplitude modulation–to–phase modulation (AM/PM) effects, which ultimately degrade radar performance. Consequently, suppressing envelope fluctuation is a critical requirement in OFDM radar waveform design.

Unlike conventional communication-oriented OFDM design, radar waveform synthesis is subject to additional system-driven constraints on subcarrier weights. In practical radar applications, subcarrier coefficients are often restricted by multipath avoidance^10^, hardware limitations^11^, and target-oriented illumination^12^ strategies. These requirements limit the feasible multicarrier coefficient space and prevent arbitrary adjustment of subcarrier weights. As a result, envelope behavior can no longer be independently controlled and instead becomes an implicit consequence of constrained multicarrier coefficient allocation. Consequently, in practical OFDM radar systems where subcarrier characteristics are restricted, envelope fluctuation suppression becomes a constrained multicarrier coefficient design problem rather than an unconstrained waveform optimization problem.

In existing OFDM radar waveform design, envelope fluctuation suppression is typically incorporated into the waveform optimization process. One class of approaches directly optimizes waveform samples while incorporating envelope-related metrics. Various metrics have been adopted for this purpose. The peak-to-average-power ratio (PAR) constrains envelope peaks^13^, while constant-modulus (CM) constraints enforce strict amplitude uniformity^14,15^. Other dynamic-range-based metrics, such as peak-to-valley-power ratio (PVR)^16,17^ and dynamic range ratio (DRR)^18^, have also been used to characterize envelope variation. More recently, the coefficient of variance of envelope (CVE)^19^ has been proposed to evaluate envelope fluctuation over all time instants. Unlike extreme-value-based metrics, e.g., PAR, PVR and DRR, the calculation of CVE covers all time instants, and any local increase in envelope fluctuation leads to an increase in CVE, providing a global characterization of envelope smoothness. The three metrics are compared in the next section by employing a high-power amplifier (HPA) model.

Despite these developments, most existing studies consider envelope fluctuation suppression without explicitly accounting for subcarrier constraints. However, subcarrier characteristics play a fundamental role in OFDM radar waveform performance. For instance, subcarrier power allocation directly affects the ambiguity function and sidelobe behavior^20,21^. In many practical designs, subcarrier weights may be partially fixed, bounded, or required to satisfy power distribution uniformity. Under such constraints, minimizing envelope fluctuation while preserving desired spectrum characteristics becomes a coupled optimization problem. Conventional unconstrained envelope control strategies are therefore not directly applicable.

Motivated by these observations, this work investigates envelope fluctuation suppression under explicit subcarrier constraints. The objective is to develop a multicarrier coefficient design mechanism that achieves envelope smoothing while preserving prescribed subcarrier characteristics. To this end, we adopt the metric CVE as the optimization metric and introduce a modified formulation that facilitates analytical simplification under energy constraints in OFDM radar waveform design. The resulting problem is reformulated as an $\mathrm {L_1}$-norm maximization problem, for which an iterative solution approach is developed. The main contributions of this paper are summarized as follows:

1. CVE-based envelope control: We adopt the metric CVE as the optimization metric. This allows us to achieve consistent suppression of envelope fluctuations over all time instants. Unlike extreme-value-based metrics such as PAR or PVR, CVE provides a global measure of envelope smoothness. It avoids overlooking non-extreme but distributed fluctuations.
2. Subcarrier-constrained waveform formulation: The CVE-based optimization is formulated under explicit subcarrier constraints, including a total power constraint and subcarrier power ratio (SPR) constraints. This formulation ensures that envelope smoothing is achieved without implicitly reshaping the subcarrier power distribution, thereby enabling controllable and interpretable regulation of spectral flatness and preserving radar-relevant waveform characteristics.
3. Efficient algorithm via complex $\mathrm {L_1}$-principal component analysis ($\mathrm {L_1}$-PCA): By exploiting the structure of complex $\mathrm {L_1}$-PCA, the proposed problem is reformulated into an equivalent surrogate form that admits an efficient iterative solution. A complete algorithm is developed, together with theoretical analysis on its optimality conditions and convergence behavior.

The remainder of this work is structured as follows: Section II introduces the OFDM signal model and reviews related research. Section III presents the proposed method and discusses its characteristics in detail. Section IV provides simulations of the proposed methods and compares them with existing approaches. Finally, Section V concludes the paper.

Notations: In this work, bold lowercase letters (e.g., $\boldsymbol{x}$), bold uppercase letter (e.g., $\boldsymbol{X}$) represent vectors and matrices respectively. The superscripts $\left( \cdot \right) ^*, \left( \cdot \right) ^T, \left( \cdot \right) ^H$ denote conjugation, transpose and Hermitian transpose. $\odot$ and $\circledast$ denote element-wise multiplication and cyclic convolution for two vectors. $\emptyset$ is the null set. $\mathbb {R}^N$, $\mathbb {C}^N$ represent the set of *N*-dimensional real vectors and complex vectors respectively. Let $\mathbb {U}^N$ represent a set composed of all *N*-dimensional unimodular complex vectors, $\mathbb {U}^N \triangleq \left\{ \boldsymbol{a} \vert \boldsymbol{a} \in \mathbb {C}^N, \vert a_n \vert = 1, n=1,2,\ldots ,N \right\}$, while $\mathbb {C}^{M \times N}$ is the set of $M \times N$ complex matrices. $\Re {\left\{ x\right\} }$ and $\Im {\left\{ x\right\} }$ represent the real and imaginary parts of the complex variable *x*. $\Vert \cdot \Vert _{p}$ represents the *p*-norm of a vector. If there is a complex vector $\boldsymbol{x}=\left[ x_1, x_2, \ldots , x_N\right] ^T\in \mathbb {C}^N$, then define $\vert \boldsymbol{x} \vert _{\operatorname {mag}}\triangleq \left[ \vert x_1 \vert , \vert x_2 \vert , \ldots , \vert x_{N} \vert \right] ^T$, and $[\boldsymbol{x}]_n \triangleq x_n$ represents the *n*-th element of $\boldsymbol{x}$. If $\boldsymbol{a} \in \mathbb {C}^N$, define operator $\operatorname {sgn}\left( \boldsymbol{a}\right) \triangleq \left[ \frac{a_1}{\vert a_1 \vert }, \frac{a_2}{\vert a_2 \vert },\ldots , \frac{a_{M}}{\vert a_{M} \vert }\right] ^T$. $\operatorname {Diag}\left( \cdot \right)$ constructs a diagonal matrix with the given vector as its diagonal elements. $\operatorname {Tr}(\cdot )$ denotes the matrix trace operation.

## Preliminaries

### The signal model

In this study, a discrete OFDM signal model is employed for digital processing, which is derived by sampling the corresponding analog OFDM waveform. To accurately capture the fluctuations of the analog signal, oversampling is necessary^22^. Consider an OFDM waveform composed of *N* subcarriers, with their weights denoted as $\boldsymbol{x}=\left[ {x_{1},x_{2},\ldots ,x_{N}}\right] ^T\in \mathbb {C}^N$. Assume the analog signal is sampled at a rate of ${O_sN}{\Delta {f}}$, with ${O_s}$ representing the oversampling factor and $\Delta {f}$ the subcarrier spacing, the resulting sampled sequence is denoted as $\boldsymbol{y}=\left[ {y_{1}, y_{2},\ldots , y_{O_sN}}\right] ^T\in \mathbb {C}^{O_sN}$. The sequence $\boldsymbol{y}$ can also be obtained from $\boldsymbol{x}$ via an inverse discrete Fourier transform (IDFT)^23^,

1 $$\begin{aligned} {\boldsymbol{y}}=\boldsymbol{W} \boldsymbol{x}, \end{aligned}$$

where $\boldsymbol{W}\in \mathbb {C}^{O_{s}N \times N}$ denotes the first *N* columns of the $O_{s}N$-point IDFT matrix, of which the $\left( m,n\right)$-th element is defined as

2 $$\begin{aligned} \boldsymbol{W}_{m,n} = \frac{1}{O_sN} \exp \left( j\frac{2\pi (m-1)(n-1)}{O_sN}\right) . \end{aligned}$$

To accommodate the scenario where the weights of certain subcarriers are pre-assigned, the set of subcarrier indices is partitioned into two subsets, $S_{v}=\{v_1,v_2,\ldots ,{v_{N_{v}}}\}$ and $S_{t}=\left\{ t_1,t_2,\ldots ,r_{N_{t}}\right\}$, corresponding to non-adjustable subcarriers and adjustable subcarriers, respectively, ${N_v}$ and $N_{t}$ are the number of subcarriers in each set. Obviously, $S_{v} \cup S_{t} = \left\{ 1, 2, \ldots , N\right\}$. The weights of subcarriers indexed by $S_{v}$ and $S_{t}$ are denoted as $\boldsymbol{x}_{v} \in \mathbb {C}^{N_{v}}$ and $\boldsymbol{x}_{t}\in \mathbb {C}^{N_{t}}$ respectively. Therefore, $\boldsymbol{y}$ can also be expressed as,

3 $$\begin{aligned} \begin{aligned} \boldsymbol{y}&= \boldsymbol{W}_{v}\boldsymbol{x}_{v} + \boldsymbol{W}_{t} \boldsymbol{x}_{t}\\&=\boldsymbol{y}_{v} + \boldsymbol{W}_{t} \boldsymbol{x}_{t}, \end{aligned} \end{aligned}$$

where $\boldsymbol{W}_{v}$ consists of the columns of $\boldsymbol{W}$ indexed by $S_{v}$, while $\boldsymbol{W}_{t}$ consists of the columns indexed by $S_{t}$.

### Analysis of envelope fluctuation metrics

Based on the signal model, this subsection provides the definitions of several metrics for evaluating envelope fluctuation. Among them, PAR, as defined in^24^, is given by

4 $$\begin{aligned} \operatorname {PAR}\left( \boldsymbol{y} \right) \triangleq \frac{\max _{p=1,2,\ldots ,O_sN} \vert \left[ \boldsymbol{y} \right] _p \vert ^2}{\frac{1}{O_s N} \sum _{p=1}^{O_s N} \vert \left[ \boldsymbol{y} \right] _p \vert ^2 }. \end{aligned}$$

PVR, as defined in^17^, is given by

5 $$\begin{aligned} \operatorname {PVR}\left( \boldsymbol{y} \right) \triangleq \frac{\max _{p=1,2,\ldots ,O_sN} \vert \left[ \boldsymbol{y} \right] _p \vert ^2}{\min _{p=1,2,\ldots ,O_sN} \vert \left[ \boldsymbol{y} \right] _p \vert ^2}. \end{aligned}$$

CVE, as defined in^19^, is given by

6 $$\begin{aligned} \operatorname {CVE}_{\textrm{mean}}({\boldsymbol{y}}) \triangleq \frac{\frac{1}{O_s N} \sum _{p=1}^{O_{s}N}{\left( \vert \left[ \boldsymbol{y} \right] _p \vert - \bar{\beta } \right) ^2}}{\bar{\beta }^2}, \quad \bar{\beta } = \frac{1}{O_s N}\sum _{p=1}^{O_s N} \vert \left[ \boldsymbol{y} \right] _p \vert . \end{aligned}$$

Here, the subscript ”$\textrm{mean}$” indicates that the CVE is calculated with respect to the envelope mean. Based on these metrics, a low envelope fluctuation waveform design can be formulated as:

7 $$\begin{aligned} \mathscr {P}_{\texttt {1}}:\min _{\boldsymbol{x}_{t} \in \mathbb {C}^{N_{t}}} f(\boldsymbol{y}), \quad \text {with } f(\boldsymbol{y}) \in \{\operatorname {PAR}(\boldsymbol{y}),\operatorname {PVR}(\boldsymbol{y}),\operatorname {CVE}_{\textrm{mean}}(\boldsymbol{y})\},\quad s.t. \ \boldsymbol{y}=\boldsymbol{y}_{v} + \boldsymbol{W}_{t} \boldsymbol{x}_{t}. \end{aligned}$$

$\mathscr {P}_{\texttt {1}}$ can typically be solved using iterative methods. Such as the iterative least squares (ILS) approach reported in^19^.

**Fig. 1:**
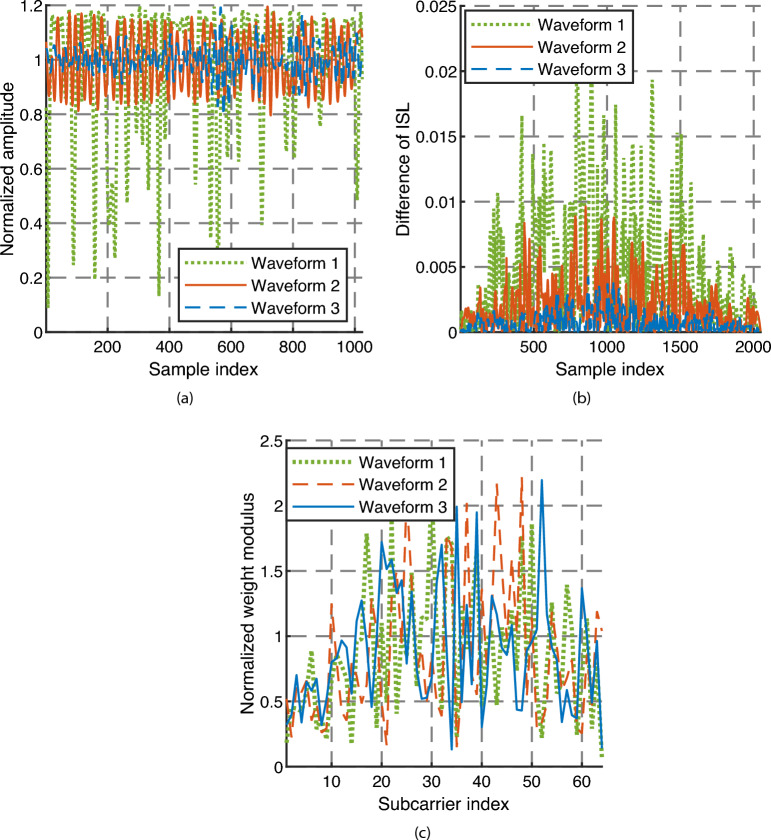
**a** Illustrative examples of OFDM waveform envelopes used to demonstrate the differences among PAR, PVR, and CVE. These waveforms are constructed for explanatory purposes only and do not correspond to the proposed method or any existing optimization algorithms; **b** Difference in auto-correlation function before and after amplification; **c** Magnitudes of the subcarrier coefficients corresponding to the three illustrative multicarrier waveform examples.

To compare different envelope fluctuation metrics, a nonlinear HPA model is employed, whose parameters are specified in^25^. Three OFDM waveform examples with identical total energy, bandwidth, and subcarrier weight settings are used as inputs to the model. Among them, all waveforms share the same PAPR, while Waveforms 2 and 3 additionally exhibit identical PVR. However, their CVE values are different: Waveform 1 has the largest CVE, whereas Waveform 3 has the smallest. Figure 1a illustrates the temporal envelope behaviors of the three waveforms. Figure 1b depicts the difference of autocorrelation functions (ACFs) before and after amplification. The observed differences primarily arise from nonlinear distortion introduced by the HPA. The results suggest that, under identical peak-related constraints, reducing CVE tends to mitigate nonlinear distortion after amplification.

In addition, the uniformity of the subcarrier power distribution is observed to be unsatisfactory, as illustrated in Fig. 1c. Specifically, a larger portion of energy is allocated to subcarriers near the center of the bandwidth than to those at the edges, whereas subcarriers at the spectral edges receive significantly lower energy. This phenomenon highlights the necessity of incorporating subcarrier-related constraints to preserve a controlled spectrum structure, which is explicitly addressed in the next section. To evaluate the uniformity of subcarrier power distribution, we introduce the subcarrier power ratio (SPR) metric. As defined in^26^, SPR is given by

8 $$\begin{aligned} \operatorname {SPR}\left( \boldsymbol{x}_{t}\right) = \frac{\max _{n=1,2,\ldots ,N_{t}}{\vert \left[ \boldsymbol{x}_{t}\right] _n \vert ^2}}{\frac{1}{N_{t}} \sum _{n=1}^{N_{t}}{\vert \left[ \boldsymbol{x}_{t}\right] _n\vert ^2}}, \end{aligned}$$

SPR reflects the ratio between the peak and average subcarrier power in the frequency domain, similar to the PAR defined in the time domain. As the SPR decreases, the power distribution becomes more uniform. When $\operatorname {SPR}=1$, all subcarrier weights are assigned equal magnitude.

### $\mathrm {L_1}$-PCA and optimization techniques

The following derivation involves optimizing the $\mathrm {L_1}$-norm for complex data. This approach, known as complex $\mathrm {L_1}$-principal component analysis ($\mathrm {L_1}$-PCA), has been extensively studied in^27^. Several relevant conclusions are reviewed here.

For the purposes of the following derivation, we introduce a computational approach for the operator $\operatorname {sgn}\left( \cdot \right)$

9 $$\begin{aligned} \operatorname {sgn}\left( \boldsymbol{a}\right) = \mathop {\arg \max }_{\boldsymbol{b} \in \mathbb {U}^{M}}{\Re \{\boldsymbol{b}^H \boldsymbol{a}\}}. \end{aligned}$$

The complex $\mathrm {L_1}$-PCA method shows that, for a given matrix $\boldsymbol{X} \in \mathbb {C}^{N \times M}$, the problem of finding a complex vector $\boldsymbol{q}\in \mathbb {C}^{N}$ that maximizes $\Vert \boldsymbol{q}^H \boldsymbol{X} \Vert _1$ is equivalent to finding a vector $\boldsymbol{b} \in \mathbb {U}^{M}$ such that $\Vert \boldsymbol{X} \boldsymbol{b} \Vert _2$ is maximized, i.e.,

10 $$\begin{aligned} \begin{aligned} \max _{\boldsymbol{q} \in \mathbb {C}^{N}, \boldsymbol{q}^H \boldsymbol{q} = 1} \Vert \boldsymbol{q}^H \boldsymbol{X} \Vert _1 = \max _{\boldsymbol{b} \in \mathbb {U}^{M}} \Vert \boldsymbol{X} \boldsymbol{b} \Vert _2. \end{aligned} \end{aligned}$$

The detailed derivation can be found in^27^. As stated in the reference, the $\mathrm {L_1}$-PCA problem is formally NP-hard and challenging to resolve. After transforming it into a unimodular quadratic maximization (UQM) problem, the reference provides an iterative method for obtaining a local optimum, where $\boldsymbol{b}$ is iteratively updated as follows,

11 $$\begin{aligned} \boldsymbol{b}^{(k+1)} = \operatorname {sgn}\left( \boldsymbol{A}_d \boldsymbol{b}^{(k)}\right) , \end{aligned}$$

where $\boldsymbol{A}_d = \boldsymbol{X}^H \boldsymbol{X} - \operatorname {Diag} \left( \left[ \Vert \textbf{x}_{1}\Vert ^{2}_{2}, \Vert \textbf{x}_{2}\Vert ^{2}_{2}, \ldots , \Vert \textbf{x}_{M}\Vert ^{2}_{2}\right] ^T\right)$, $\textbf{x}_{m}$ is the *m*-th column vector of $\boldsymbol{X}$.

## The proposed scheme

### The basic method

As discussed above, subcarrier constraints may play an important role in radar performance. Next, we consider the optimization of $\mathscr {P}_{\texttt {1}}$ incorporated with subcarrier constraints. When the waveform is assumed to operate with HPAs in the saturation region, such as in some radar systems, local envelope deviations at any time instant may result in nonlinear distortion. This motivates the need for overall envelope fluctuation suppression. The metric CVE is more effective in capturing distributed envelope variations. Therefore, it is adopted as the sole optimization metric for subsequent derivation. The problem is formulated as follows:

12 $$\begin{aligned} \begin{aligned} \mathscr {P}_{\texttt {2}}:\min _{\boldsymbol{x}_{t} \in \mathbb {C}^{N_{t}}}&\frac{\frac{1}{O_s N} \sum _{p=1}^{O_{s}N}{\left( \vert \left[ \boldsymbol{y}\right] _p \vert - \bar{\beta } \right) ^2}}{\beta ^2} \\ \text {s.t.} \quad&C_{0}:\boldsymbol{y} =\boldsymbol{y}_{v}+\boldsymbol{W}_{t} \boldsymbol{x}_{t}\\&C_{1}:\bar{\beta } = \frac{1}{O_s N}\sum _{l=1}^{O_s N} \vert \left[ \boldsymbol{y}\right] _l \vert \\&C_{2}:\operatorname {SPR}\left( \boldsymbol{x}_{t}\right) \le \eta \\&C_{3}:\sum _{n=1}^{N_{t}}{\vert \left[ \boldsymbol{x}_{t}\right] _n\vert ^2} = {P}_{t}, \end{aligned} \end{aligned}$$

Constraint $C_{2}$ limits the degree of subcarrier power concentration through parameter $\eta$. When $\eta = 1$, all subcarriers have equal power, whereas larger $\eta$ values allow increasing flexibility. In the limit of large $\eta$, $C_{2}$ becomes inactive and $\mathscr {P}_{\texttt {2}}$ reduces to unconstrained CVE minimization. Constraint $C_3$ enforces the squared-modulus sum of all adjustable subcarrier weights to be a constant $P_{t}$, effectively serving as a total energy constraint, this can demonstrate by Parseval’s theorem. Given that $\sum _{n=1}^{N_{v}}{\vert \left[ \boldsymbol{x}_{v}\right] _n\vert ^2} = P_{v}$, and ${P}_{all} = {P}_{v} + {P}_{t}$, the total energy is13The newly introduced constraints make Problem $\mathscr {P}_{\texttt {2}}$ challenging to solve using the ILS algorithm. To facilitate subsequent optimization, the original mean-based evaluation is reformulated into an root mean square (RMS)-based expression. Under the total power constraint $C_3$, the time domain envelope energy is fixed over the feasible set. As a result, the mean-based and RMS-based CVE formulations are consistent in penalizing envelope fluctuations across time instants and yield similar envelope fluctuation trends. The RMS-based expression further admits a quadratic representation, which enables problem simplification and facilitates the subsequent optimization. The new definition is given by

14 $$\begin{aligned} \begin{aligned} \operatorname {CVE}_{\textrm{rms}}(\boldsymbol{y}) \triangleq \frac{ \frac{1}{O_s N} \sum _{p=1}^{O_{s}N}{ \left[ \vert \left[ \boldsymbol{y}\right] _p \vert - \beta \right] ^2 }}{\beta ^2}, \quad \beta = \sqrt{\frac{1}{O_s N}\sum \limits _{p=1}^{O_{s}N}{\vert \left[ \boldsymbol{y}\right] _p \vert ^{2}}}. \end{aligned} \end{aligned}$$

Compared to $\operatorname {CVE}_{\textrm{mean}}$, $\operatorname {CVE}_{\textrm{rms}}$ uses the root mean square of the envelope as a benchmark. Both metrics reach their minimum when the envelope is constant. Under the fixed total energy constraint, $\beta = \frac{\sqrt{{P}_{\textrm{all}}}}{O_sN}$ becomes a constant and can therefore be omitted from the expression. Meanwhile, the SPR constraint can be equivalently rewritten as

15 $$\begin{aligned} \max _{n=1,2,\ldots ,N_{t}}{\vert \left[ \boldsymbol{x}_{t}\right] _n \vert ^2} \le \frac{\eta {P}_{t}}{{N}_{t}} \Longleftrightarrow \vert \left[ \boldsymbol{x}_{t}\right] _n \vert \le \sqrt{\frac{\eta {P}_{t}}{{N}_{t}}}, \ \forall n = 1,2,\ldots ,N_{t}. \end{aligned}$$

For brevity, denote $\sqrt{\frac{\eta {P}_{t}}{{N}_{t}}} = x_{max}$.

The newly defined metric $\operatorname {CVE}_{\textrm{rms}}$ and the simplified SPR constraints are substituted into $\mathscr {P}_{\texttt {2}}$. Constant terms that do not affect the optimization are then discarded. As a result, a surrogate formulation of $\mathscr {P}_{\texttt {2}}$ is obtained as

16 $$\begin{aligned} \begin{aligned} \mathscr {P}_{\texttt {3}}:\min _{\boldsymbol{x}_{t} \in \mathbb {C}^{N_{t}}}&\sum _{p=1}^{O_{s}N} {\left( \vert \left[ \boldsymbol{y}\right] _p \vert - \frac{\sqrt{{P}_{\textrm{all}}}}{O_sN} \right) ^2 }\\ \text {s.t.} \quad&C_{0}:\boldsymbol{y} =\boldsymbol{y}_{v}+\boldsymbol{W}_{t} \boldsymbol{x}_{t}\\&C_{2}:\vert \left[ \boldsymbol{x}_{t}\right] _n \vert \le x_{max}, \ \forall n = 1,2,\ldots ,N_{t} \\&C_{3}:\Vert \boldsymbol{x}_{t}\Vert _2^2 = {P}_{t}.\\ \end{aligned} \end{aligned}$$

In $\mathscr {P}_{\texttt {3}}$, constraint $C_1$ is omitted since $\beta$ is a constant. Next, the objective function is further simplified.

17 $$\begin{aligned} \begin{aligned} \sum \limits _{l=1}^{O_{s}N}{ \left( \vert { \left[ \boldsymbol{y}\right] _l} \vert - \frac{\sqrt{{P}_{all}}}{O_sN} \right) ^2 }&= \left[ \boldsymbol{y} - \frac{\sqrt{{P}_{all}}}{O_sN} \cdot \operatorname {sgn}(\boldsymbol{y}) \right] ^H \left[ \boldsymbol{y} - \frac{\sqrt{{P}_{all}}}{O_sN} \cdot \operatorname {sgn}(\boldsymbol{y}) \right] \\&= \frac{2}{O_sN} {P}_{all} - \frac{2\sqrt{{P}_{all}}}{O_sN} \Vert \boldsymbol{y} \Vert _1. \end{aligned} \end{aligned}$$

Equation (17) indicates that minimizing $\operatorname {CVE}_{\textrm{rms}}(\boldsymbol{y})$ in $\mathscr {P}_{\texttt {3}}$ is equivalent to maximizing $\Vert \boldsymbol{y}\Vert _1$. To facilitate the derivation, we temporarily relax the constraints on subcarrier weight magnitudes by assuming $x_{max}=+\infty$ and setting $P_{t}$ to unity, $\mathscr {P}_{3}$ is simplified as follows:

18-1 $$\begin{aligned} \mathscr {P}_{\texttt {4}}:\max _{\boldsymbol{x}_{t} \in \mathbb {C}^{N_{t}}}&\Vert \boldsymbol{y} \Vert _1 \end{aligned}$$

18-2 $$\begin{aligned} \text {s.t.} \quad&C_{0}: \boldsymbol{y} = \boldsymbol{W}_{t} \boldsymbol{x}_{t} + \boldsymbol{y}_{v} \end{aligned}$$

18-3 $$\begin{aligned}&C_{3}: \Vert \boldsymbol{x}_{t}\Vert _2^2 = 1 . \end{aligned}$$

For applying results from complex $\mathrm {L_1}$-PCA, $\mathscr {P}_{4}$ is reformulated step by step in next. First, the $\mathrm {L_1}$-norm of a complex vector is rewritten using the sign operator. Specifically, for any complex vector $\boldsymbol{x}$, its $\mathrm {L_1}$-norm can be expressed as $\Vert \boldsymbol{x} \Vert _1 = \operatorname {Tr} \left\{ \operatorname {sgn}(\boldsymbol{x})\boldsymbol{x}^H\right\}$. Introducing the trace operator here does not change the value of the expression, but allows a unified matrix-form representation,

19 $$\begin{aligned} \begin{aligned}&\max _{\boldsymbol{x}_{t} \in \mathbb {C}^{N_{t}}, \Vert \boldsymbol{x}_{t}\Vert _2^2 = 1} \Vert \boldsymbol{x}_{t}^H \boldsymbol{W}_{t}^H + \boldsymbol{y}_{v}^H \Vert _1 \\ =&\max _{\boldsymbol{x}_{t} \in \mathbb {C}^{N_{t}}, \Vert \boldsymbol{x}_{t}\Vert _2^2 = 1} \operatorname {Tr} \Big \{ \operatorname {sgn} \big ( \boldsymbol{W}_{t} \boldsymbol{x}_{t} + \boldsymbol{y}_{v} \big ) \big ( \boldsymbol{x}_{t}^H \boldsymbol{W}_{t}^H + \boldsymbol{y}_{v}^H \big ) \Big \} \end{aligned} \end{aligned}$$

Next, an auxiliary unimodular variable $\boldsymbol{b}\in \mathbb {U}^{O_sN}$ is introduced. According to the property of the sign operator, the projection of term $(\boldsymbol{W}_{t} \boldsymbol{x}_{t} + \boldsymbol{y}_{v})^H$ onto $\boldsymbol{b}$ is maximized if and only if $\boldsymbol{b}=\operatorname {sgn}(\boldsymbol{W}_{t} \boldsymbol{x}_{t} + \boldsymbol{y}_{v})$, (19) can be written as follows,

20 $$\begin{aligned} \begin{aligned} \max _{\begin{array}{c} \boldsymbol{x}_{t} \in \mathbb {C}^{N_{t}},\Vert \boldsymbol{x}_{t}\Vert _2^2 = 1,\\ \boldsymbol{b} \in \mathbb {U}^{O_s N} \end{array}}{\Re \Big \{ \operatorname {Tr} \left\{ \boldsymbol{b} \boldsymbol{x}_{t}^H \boldsymbol{W}_{t}^H + \boldsymbol{b} \boldsymbol{y}_{v}^H \right\} \Big \} } \end{aligned} \end{aligned}$$

Since the trace of a scalar is the scalar itself, and the real-part operator commutes with the trace, the order of the $\operatorname {Tr}\{\cdot \}$ and $\Re \{\cdot \}$ operators does not affect the result. By exploiting the cyclic property of the trace, $\boldsymbol{b}$ can be moved to the rightmost position, after which the trace operator can be omitted, (20) can be rewritten as,

21 $$\begin{aligned} \begin{aligned} \max _{\boldsymbol{b}\in \mathbb {U}^{O_sN}}{\max _{\boldsymbol{x}_{t}\in \mathbb {C}^{N_{t}},\Vert \boldsymbol{x}_{t}\Vert _2^2 = 1}{\Re \left\{ {\boldsymbol{x}_{t}^H\boldsymbol{W}}_{t}^H\boldsymbol{b}+\boldsymbol{y}_{v}^H\boldsymbol{b}\right\} }} \end{aligned} \end{aligned}$$

Finally, because the objective function is linear in both $\boldsymbol{x}_{t}$ and $\boldsymbol{b}$, the maximization over the two variables can be decoupled. For a fixed $\boldsymbol{b}$, the inner maximization with respect to $\boldsymbol{x}_{t}$ under the unit-norm constraint admits a closed-form solution. Therefore, (21) can be rewritten as,

22 $$\begin{aligned} \begin{aligned} \max _{\boldsymbol{b} \in \mathbb {U}^{O_s N}} {\left( \Vert \boldsymbol{W}_{t}^H \boldsymbol{b} \Vert _2+ \frac{\boldsymbol{y}_{v}^H \boldsymbol{b} + \boldsymbol{b}^H \boldsymbol{y}_{v}}{2} \right) } . \end{aligned} \end{aligned}$$

With the derivation from (19) to (22), maximizing the $\mathrm {L_1}$-norm of a linear function over a unit-energy complex vector is transformed into the maximization of a term over a unimodular complex vector. Denote the objective function in (19) and (22) as

23 $$\begin{aligned} g_1\left( \boldsymbol{x}_{t}\right) =\Vert \boldsymbol{x}_{t}^H \boldsymbol{W}_{t}^H + \boldsymbol{y}_{v}^H \Vert _1 \end{aligned}$$

and

24 $$\begin{aligned} g_2\left( \boldsymbol{b}\right) =\Vert \boldsymbol{W}_{t}^H \boldsymbol{b} \Vert _2+ \frac{\left( {\boldsymbol{y}_{v}^H\boldsymbol{b}+\boldsymbol{b}^H\boldsymbol{y}_{v}} \right) }{2}. \end{aligned}$$

Let $\boldsymbol{x}_{t}^{\left( opt\right) }$ be the solution of $g_1\left( \boldsymbol{x}_{t}\right)$, and $\boldsymbol{b}^{\left( opt\right) }$ be the solution of $g_2\left( \boldsymbol{b}\right)$, thus,

25 $$\begin{aligned} g_1\left( \boldsymbol{x}_{t}^{\left( opt\right) }\right) =g_2\left( \boldsymbol{b}^{\left( opt\right) }\right) . \end{aligned}$$

According to the derivations in (21), $\boldsymbol{x}_{t}^{\left( opt\right) }$ and $\boldsymbol{b}^{\left( opt\right) }$ are related as follows:

26 $$\begin{aligned} \boldsymbol{b}^{\left( opt\right) }=\operatorname {sgn}\left( \boldsymbol{W}_{t}\boldsymbol{x}_{t}^{\left( opt\right) }+\boldsymbol{y}_{v}\right) , \end{aligned}$$

and

27 $$\begin{aligned} \boldsymbol{x}_{t}^{\left( opt\right) }=\mathop {\arg \max }_{\boldsymbol{x}_{t}\in \mathbb {C}^{N_{t}},\Vert \boldsymbol{x}_{t}\Vert _2^2 = 1}{\Re \left\{ \boldsymbol{x}_{t}^H\boldsymbol{W}_{t}^H\boldsymbol{b}^{\left( opt\right) }\right\} }=\frac{\boldsymbol{W}_{t}^H\boldsymbol{b}^{\left( opt\right) }}{\Vert \boldsymbol{W}_{t}^H\boldsymbol{b}^{\left( opt\right) } \Vert _2} . \end{aligned}$$

Substituting (27) into (26),

28 $$\begin{aligned} \boldsymbol{b}^{\left( opt\right) }=\text {sgn}\left( \boldsymbol{W}_{t}\boldsymbol{W}_{t}^H\boldsymbol{b}^{\left( opt\right) }+{\Vert \boldsymbol{W}_{t}^H\boldsymbol{b}^{\left( opt\right) } \Vert _2}\boldsymbol{y}_{v} \right) . \end{aligned}$$

Due to the non-convex nature of the domains of ${\boldsymbol{x}}_{t}$ and $\boldsymbol{b}$, finding the global optimum is challenging, but it is possible to find a local optimum. Equations (26), (27), and (28) also hold for local maximizers of $g_2\left( \boldsymbol{b}\right)$. Denote the element phases of $\boldsymbol{b}$ as a vector $\boldsymbol{\phi }=\left[ \phi _1,\phi _2,\ldots ,\phi _{O_sN}\right] ^T\in \mathbb {R}^{O_sN}$, where ${\phi }_m \in \left[ 0,2\pi \right)$ for $m=1,2,\ldots ,O_sN$. Then, $\boldsymbol{b}= \left[ e^{j\phi _1},e^{j\phi _2},\ldots ,e^{j\phi _{O_sN}}\right] ^T$. The first and second derivatives of $g_2\left( \boldsymbol{b}\right)$ with respect to $\boldsymbol{\phi }$ are given by

29 $$\begin{aligned} \nabla g_2(\boldsymbol{\phi }) = \frac{\Im \left\{ \boldsymbol{b}^* \odot \left( \boldsymbol{W}_{t} \boldsymbol{W}_{t}^H \boldsymbol{b} + \Vert \boldsymbol{W}_{t}^H \boldsymbol{b}\Vert _2 \boldsymbol{y}_{v} \right) \right\} }{\Vert \boldsymbol{W}_{t}^H \boldsymbol{b}\Vert _2} \end{aligned}$$

and

30 $$\begin{aligned} \begin{aligned} \nabla ^2g_2\left( \boldsymbol{\phi }\right) = \frac{1}{\Vert \boldsymbol{W}_{t}^H \boldsymbol{b}\Vert _2} \Re \bigg \{\textrm{Diag}\left( \boldsymbol{b}\right) ^H\boldsymbol{W}_{t}\boldsymbol{W}_{t}^H \textrm{Diag}\left( \boldsymbol{b}\right) - \textrm{Diag}\Big [{\boldsymbol{b}^*}\odot \left( \boldsymbol{W}_{t} \boldsymbol{W}_{t}^H \boldsymbol{b} \right. \\ \left. + \Vert \boldsymbol{W}_{t}^H \boldsymbol{b}\Vert _2 \boldsymbol{y}_{v}\right) \Big ]\bigg \} -\frac{\Im \left\{ \boldsymbol{b}^*\odot \left[ \boldsymbol{W}_{t} \boldsymbol{W}_{t}^H \boldsymbol{b} \right] \right\} \left( \Im \left\{ \boldsymbol{b}^*\odot \left[ \boldsymbol{W}_{t} \boldsymbol{W}_{t}^H \boldsymbol{b} \right] \right\} \right) ^T}{\Vert \boldsymbol{W}_{t}^H \boldsymbol{b}\Vert _2^3} \end{aligned} \end{aligned}$$

Substituting (28) into (29), we obtain $\nabla g_2\left( \boldsymbol{\phi }\right) =\boldsymbol{0}$, which confirms that (28) is a necessary condition for all local maximizers. However, it is not a sufficient condition for a local maximizer, as it does not guarantee that $\nabla ^2g_2(\boldsymbol{\phi })$ is negative definite. A necessary and sufficient condition characterizing a local maximizer of the objective function $g_2\left( \boldsymbol{b}\right)$ is given in next. For notion brevity, the function $\mathscr {W}\left( \boldsymbol{b}\right) =\boldsymbol{b}^*\odot \left( \boldsymbol{W}_{t} \boldsymbol{W}_{t}^H \boldsymbol{b} + \Vert \boldsymbol{W}_{t}^H \boldsymbol{b}\Vert _2 \boldsymbol{y}_{v}\right)$ is defined previously, with its *m*-th element is denoted by $\left[ \mathscr {W}\left( \boldsymbol{b}\right) \right] _{m}$. Then, the following holds:

31 $$\begin{aligned} \mathscr {W}\left( \boldsymbol{b}\right) \in \mathbb {R}^{O_{s}N}, \left[ \mathscr {W}\left( \boldsymbol{b}\right) \right] _{m}>\Vert \boldsymbol{w}_m \Vert _2^2,m\in \left\{ 1,2,\ldots ,O_sN\right\} , \end{aligned}$$

where $\boldsymbol{w}_m$ is the *m*-th row of $\boldsymbol{W}_{t}$. The detailed proof, based on first- and second-order optimality conditions, is provided in Appendix A

Based on the above analysis, an iterative solution method is proposed. In the *k*-th iteration, $\boldsymbol{x}_{t}$ and $\boldsymbol{b}$ are updated as follows:

32 $$\begin{aligned} \boldsymbol{x}_{t}^{(k)}=\mathop {\arg \max }_{\boldsymbol{x}_{t}\in \mathbb {C}^{N_{t}},\Vert \boldsymbol{x}_{t}\Vert _2^2 = 1}{\Re \left\{ \boldsymbol{x}_{t}^H\boldsymbol{W}_{t}^H\boldsymbol{b}^{\left( k-1\right) }\right\} } , \end{aligned}$$

and

33 $$\begin{aligned} \boldsymbol{b}^{\left( k\right) }=\operatorname {sgn}\left( \boldsymbol{W}_{t}\boldsymbol{x}_{t}^{(k)}+\boldsymbol{y}_{v}\right) . \end{aligned}$$

In next, the convergence of the iterative method is proved. Substituting (32) and (33) into $g_1\left( \boldsymbol{x}_{t}\right)$ and $g_2\left( \boldsymbol{b}\right)$, respectively,

34 $$\begin{aligned} \begin{aligned}&\Vert \boldsymbol{W}_{t}^H \boldsymbol{b}^{(k+1)} \Vert _2 + \Re \left( \boldsymbol{y}_{v}^H \boldsymbol{b}^{(k+1)} \right) \longleftarrow g_2(\boldsymbol{b}^{(k+1)})\\ =&\Re \left\{ \boldsymbol{x}_{t}^{\left( k+2\right) H}\boldsymbol{W}_{t}^H\boldsymbol{b}^{\left( k+1\right) }+\boldsymbol{y}_{v}^H\boldsymbol{b}^{\left( k+1\right) }\right\} \\ \ge&\Re \left\{ \boldsymbol{x}_{t}^{\left( k+1\right) H}\boldsymbol{W}_{t}^H\boldsymbol{b}^{\left( k+1\right) }+\boldsymbol{y}_{v}^H\boldsymbol{b}^{\left( k+1\right) }\right\} \\ =&\Re \left\{ \left( \boldsymbol{x}_{t}^{\left( k+1\right) H}\boldsymbol{W}_{t}^H+\boldsymbol{y}_{v}^H\right) *\operatorname {sgn}\left( \boldsymbol{W}_{t}\boldsymbol{x}_{t}^{(k+1)}+\boldsymbol{y}_{v}\right) \right\} \longleftarrow g_1(\boldsymbol{x}_{t}^{(k+1)})\\ \ge&\Re \left\{ \left( \boldsymbol{x}_{t}^{\left( k+1\right) H}\boldsymbol{W}_{t}^H+\boldsymbol{y}_{v}^H\right) *\boldsymbol{b}^{\left( k\right) }\right\} \\ =&\Vert \boldsymbol{W}_{t}^H \boldsymbol{b}^{(k)} \Vert _2 + \Re \left( \boldsymbol{y}_{v}^H \boldsymbol{b}^{(k)} \right) \longleftarrow g_2(\boldsymbol{b}^{(k)}). \end{aligned} \end{aligned}$$

According to (34), $g_2(\boldsymbol{b}^{(k+1)}) \ge g_1(\boldsymbol{x}_{t}^{(k+1)}) \ge g_2(\boldsymbol{b}^{(k)})$. The objective functions $g_1\left( {\boldsymbol{x}}_{t} \right)$ and $g_2\left( \boldsymbol{b} \right)$ increase monotonically with each iteration, which indicates the iterative process converges. Substituting (32) into (33), a single iteration can be compactly expressed as

35 $$\begin{aligned} \boldsymbol{b}^{(k+1)} = \operatorname {sgn}\left( \boldsymbol{W}_{t} \boldsymbol{W}_{t}^H \boldsymbol{b}^{(k)} + \Vert \boldsymbol{W}_{t}^H \boldsymbol{b}^{(k)} \Vert _2 \boldsymbol{y}_v \right) . \end{aligned}$$

**Fig. 2:**
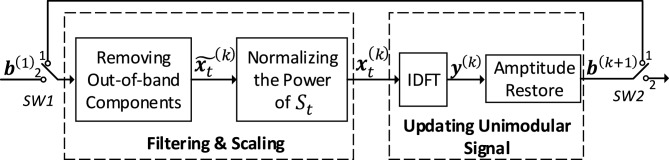
the block diagram of the Basic Method.

The iterative procedure is referred to as the Basic Method in this work. As illustrated in Fig. 2, each iteration consists of a frequency-domain filtering followed by a unimodular signal scaling, corresponding to Equations (32) and (33) respectively. The detailed steps are outlined in Algorithm 1.

**Algorithm 1:**
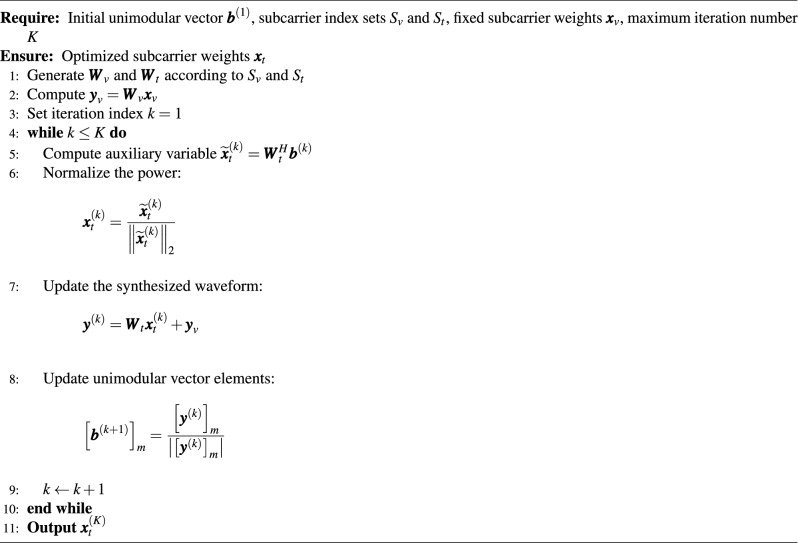
Basic PCA-L1-Based CVE Reduction Method

### Maximum magnitude constraints on subcarrier weights

In the Basic Method, the weight magnitude constraints, i.e., $C_{2}$, are temporarily relaxed. As a result, the magnitudes of weights can be still excessively high. The issue can be avoided by reintroducing $C_{2}$ constraints as follows:

36-1 $$\begin{aligned} \mathscr {P}_{\texttt {5}}:\max _{\boldsymbol{x}_{t} \in \mathbb {C}^{N_{t}}}&\Vert \boldsymbol{y} \Vert _1 \end{aligned}$$

36-2 $$\begin{aligned} \text {s.t.} \quad&C_{0}:\boldsymbol{y} = \boldsymbol{W}_{t} \boldsymbol{x}_{t} + \boldsymbol{y}_i \end{aligned}$$

36-3 $$\begin{aligned}&C_{2}:\left| \left[ \boldsymbol{x}_{t}\right] _{n} \right| \le x_{max},\quad \forall n=1,\ldots ,N_{t} \end{aligned}$$

36-4 $$\begin{aligned}&C_{3}:\Vert \boldsymbol{x}_{t}\Vert _2^2 = 1 . \end{aligned}$$

In the proposed iterative method, each iteration consists of two steps: updating the weights via (32), and updating the unimodular vector via (33). $C_{2}$ constraints can be satisfied by incorporating it into step (32) as follows:

37 $$\begin{aligned} \boldsymbol{x}_{t}^{(k)}=\mathop {\arg \max }_{\begin{array}{c} \boldsymbol{x}_{t}\in \mathbb {C}^{N_{t}},\Vert \boldsymbol{x}_{t}\Vert _2^2 = 1,\\ \Vert \boldsymbol{x}_{t} \Vert _ \infty \le x_{max} \end{array}}{\Re \left\{ \boldsymbol{x}_{t}^H\boldsymbol{W}_{t}^H\boldsymbol{b}^{\left( k-1\right) }\right\} }. \end{aligned}$$

According to Equation (9), when the cost function in (37) reaches its maximum value, $\operatorname {sgn}\left( \boldsymbol{x}_{t}^{\left( k\right) }\right) =\operatorname {sgn}\left( \boldsymbol{W}_{t}^H\boldsymbol{b}^{\left( k-1\right) }\right)$. Thus, $\boldsymbol{x}_{t}^{(k)}$ can be written as:

38 $$\begin{aligned} \boldsymbol{x}_{t}^{(k)}=\vert {\boldsymbol{x}_{t}}^{(k)}\vert _{\operatorname {mag}} \odot \operatorname {sgn}\left( \boldsymbol{W}_{t}^H\boldsymbol{b}^{\left( k-1\right) }\right) . \end{aligned}$$

The operator $\vert \cdot \vert _{\operatorname {mag}}$ is defined in Notations. Since $\operatorname {sgn}\left( \boldsymbol{W}_{t}^H\boldsymbol{b}^{\left( k-1\right) }\right)$ is known, Problem (37) is transformed into finding appropriate $\vert {\boldsymbol{x}_{t}}^{(k)}\vert _{\operatorname {mag}}$ as follows:

39 $$\begin{aligned} \vert {\boldsymbol{x}_{t}}^{(k)}\vert _{\operatorname {mag}}=\mathop {\arg \max }_{\begin{array}{c} \boldsymbol{p}\in \mathbb {R}^{N_{t}}_{+}, \left\| \boldsymbol{p} \right\| _2^2=1,\\ \left\| \boldsymbol{p} \right\| _ \infty \le x_{max} \end{array}}{\left| {\boldsymbol{W}_{t}^H\boldsymbol{b}^{\left( k-1\right) }}\right| _{\operatorname {mag}}^T\boldsymbol{p}}. \end{aligned}$$

Note that the equality constraint $\left\| \boldsymbol{p} \right\| _2^2=1$ can be relaxed to $\left\| \boldsymbol{p} \right\| _2^2 \le 1$ without affecting the optimal solution. This is because the objective function is linear in $\boldsymbol{p}$ and the feasible set is compact. Moreover, since $\boldsymbol{p}\in \mathbb {R}^{N_{t}}_{+}$ and $\left| {\boldsymbol{W}_{t}^H\boldsymbol{b}^{\left( k-1\right) }}\right| _{\operatorname {mag}}\in \mathbb {R}^{N_{t}}_{+}$ , the objective is monotonically increasing with respect to $\boldsymbol{p}$. As a result, any feasible point with $\left\| \boldsymbol{p} \right\| _2^2 < 1$ cannot be optimal, and the optimum is always attained on the boundary $\left\| \boldsymbol{p} \right\| _2^2=1$. Therefore, the relaxed problem yields the same optimal solution as the original one. Consequently, the maximization problem in (39) can be relaxed to a convex optimization problem,

40 $$\begin{aligned} \vert {\boldsymbol{x}_{t}}^{(k)}\vert _{\operatorname {mag}}=\mathop {\arg \max }_{\begin{array}{c} \boldsymbol{p}\in \mathbb {R}^{N_{t}}_{+}, \left\| \boldsymbol{p} \right\| _2^2\le 1,\\ \left\| \boldsymbol{p} \right\| _ \infty \le x_{max} \end{array}}{\left| {\boldsymbol{W}_{t}^H\boldsymbol{b}^{\left( k-1\right) }}\right| _{\operatorname {mag}}^T\boldsymbol{p}}. \end{aligned}$$

Problem (40) can be resolved using a gradient descent method, the detail steps are outlined in Algorithm 2.

**Algorithm 2:**
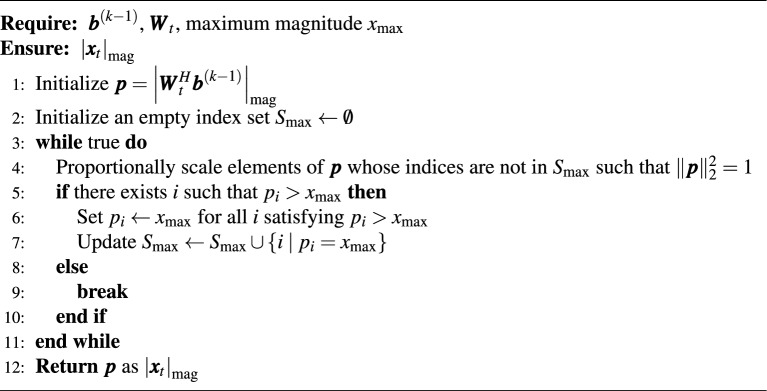
Gradient-Based Procedure for Solving Problem (40)

Substituting the obtained $\left| {\boldsymbol{x}_{t}}^{\left( k\right) }\right| _{\operatorname {mag}}$ into Eq. (38) yields the solution to (37). $\mathscr {P}_{\texttt {5}}$ can be solved by alternately performing the operations in (33) and (37). The convergence of this iterative process can be proven in a manner similar to the procedure outlined in Eq. (34).

### Discussion of the initial point

In section III, multiple constraints are imposed on the magnitudes of the weights, which, however, introduce additional computational overhead. In this section, a simple approach is proposed to roughly control the magnitudes of weights by analyzing the influence of the initial point on the resulting subcarrier power allocation in the Basic Method. Two factors of the initial inputs are investigated: the values of the initial input and the positions of $\boldsymbol{x}_i$ subcarriers .

The input and output of the Basic Method can be expressed as the following relationship:

41 $$\begin{aligned} \boldsymbol{b}^{\left( out\right) }=\boldsymbol{h}\odot \boldsymbol{b}^{\left( in\right) }, \end{aligned}$$

where ${\boldsymbol{b}}^{(in)}\in \mathbb {U}^{O_sN}$ and ${\boldsymbol{b}}^{(out)}\in \mathbb {U}^{O_sN}$ represent the input and output, respectively. $\boldsymbol{h}=\left[ e^{j \Delta \psi _1},e^{j \Delta \psi _2},\ldots ,e^{j \Delta \psi _{O_sN}} \right] ^T$, $\Delta \psi _{p}$ represents the phase rotation of the *p*-th element before and after optimization. Equation (41) suggests that the output signal can be regarded as the input signal multiplied by a phase noise (PN) term. Therefore, the impact of the initial point on the optimization result can be assessed by analyzing the characteristics of this PN term. Let $\Delta \boldsymbol{\psi }\in [-\pi ,\pi )^{O_sN}$ denote the cumulative phase rotation of $\boldsymbol{b}$ acrross all iterations, i.e.,

42 $$\begin{aligned} \Delta \boldsymbol{\psi } = \sum _k{\Delta \boldsymbol{\psi }}^{(k)}, \end{aligned}$$

with ${\Delta \boldsymbol{\psi }}^{\left( k\right) }=\left[ {\Delta \psi _1^{\left( k\right) }},{ \Delta \psi _2^{\left( k\right) }},\ldots ,{\Delta \psi _{O_sN}^{\left( k\right) }} \right] ^T$ representing the phase rotation in the *k*-th iteration. In Fig. 2, each update of $\boldsymbol{y}$ can be interpreted as a combination of low-pass filtering (removing the out-of-band components) and amplitude normalization. In time domain, the low-pass filtering is equivalent to convolution with a sinc function. If we treat the sinc function as a finite-length weighted window, then the updated $\Delta \psi _p^{(k+1)}$ only depends on the *p*-th element of $\boldsymbol{b}^{(k)}$ and its neighboring elements. Furthermore, the convergence of the proposed method implies that as $k \rightarrow \infty ,\Delta \psi _n^{(k)} \rightarrow 0$. Based on the above analysis, we make a reasonable assumption that $\Delta \psi _n^{(k)}$ are weakly dependent over *k* and have zero mean. Under this assumption, the central limit theorem for weakly dependent sequences suggests that, as *k* increases, $\Delta \boldsymbol{\psi }$ may exhibit approximately Gaussian behavior. Consequently, the aggregate term $\boldsymbol{h}$ can be modeled as a Gaussian phase noise (PN) process. A rigorous verification of the weak dependence conditions required by the central limit theorem is beyond the scope of this paper. Instead, Monte Carlo simulations are provided to empirically illustrate that the distribution of $\Delta \boldsymbol{\psi }$ is well approximated by a Gaussian distribution in the considered scenarios, thereby supporting the validity of the adopted assumption in practice.

Let $\boldsymbol{f}_{\boldsymbol{b}}$ represents the spectrum of $\boldsymbol{b}$, the *n*-th $\left( n=1, 2,\ldots , O_{s}N\right)$ element of $\boldsymbol{f}_{\boldsymbol{b}}$ is given by,

43 $$\begin{aligned} \begin{aligned} \left[ \boldsymbol{f}_{{\boldsymbol{b}}^{\left( out\right) }}\right] _n&=\sum _{m=1}^{O_s N} e^{-j \frac{2\pi }{O_s N} \left( n-1 \right) \left( m-1 \right) } \left[ \boldsymbol{b}^{\left( out\right) }\right] _{m} \\&=\sum _{m=1}^{O_s N} e^{-j \frac{2\pi }{O_s N} \left( n-1 \right) \left( m-1 \right) } e^{j \Delta \psi _ n} \left[ \boldsymbol{b}^{\left( in\right) }\right] _{m} \\&= \left[ e^{j \Delta \psi _1}, e^{j \Delta \psi _2}, \ldots , e^{j \Delta \psi _{O_s N}}\right] \cdot \begin{bmatrix}\left[ \boldsymbol{b}^{\left( in\right) }\right] _{1} \\ e^{-j \frac{2 \pi \left( n-1 \right) }{O_s N}} \left[ \boldsymbol{b}^{\left( in\right) }\right] _{2} \\ \vdots \\ e^{-j \frac{2 \pi \left( n-1 \right) (O_s N-1)}{O_s N}} \left[ \boldsymbol{b}^{\left( in \right) }\right] _{O_{s}N}\\ \end{bmatrix} \\&=\boldsymbol{h}^T \boldsymbol{z}_n, \end{aligned} \end{aligned}$$

where $\left[ \boldsymbol{z}_{n}\right] _m = e^{-j\frac{2\pi \left( n-1\right) \left( m-1\right) }{O_s N}} \left[ \boldsymbol{b}^{\left( in\right) }\right] _{m}$. By slightly transforming Eq. (43), it can be obtained that,

44 $$\begin{aligned} \begin{aligned} \left[ \boldsymbol{f}_{{\boldsymbol{b}}^{\left( out\right) }}\right] _n&= \frac{1}{O_sN}{(\boldsymbol{W}_{O_sN} \boldsymbol{h}^{*})}^{H} {\boldsymbol{W}_{O_sN} \boldsymbol{z}_k}\\&=\frac{1}{O_sN}{\boldsymbol{f}}_{{\boldsymbol{h}}^{*}}^{H}\left( {\boldsymbol{f}}_{{\boldsymbol{b}}^{\left( in\right) }}\right) _n. \end{aligned} \end{aligned}$$

In Eq. (44), the term $\left( {\boldsymbol{f}}_{{\boldsymbol{b}}^{\left( in\right) }}\right) _n$ represents the *n*-bits circularly shifted version of ${\boldsymbol{f}}_{{\boldsymbol{b}}^{\left( in\right) }}$, such that the *n*-th element $\left[ {\boldsymbol{f}}_{{\boldsymbol{b}}^{\left( in\right) }}\right] _n$ is relocated to the first position in the vector. According to^28^ and^29^, the primary energy in ${\boldsymbol{f}}_{{\boldsymbol{h}}^{*}}$ is concentrated at the zero-frequency position. Consequently, when $\left[ \boldsymbol{f}_{{\boldsymbol{b}}^{\left( in\right) }}\right] _n$ has a relatively large value, the magnitude of $\left[ \boldsymbol{f}_{{\boldsymbol{b}}^{\left( out\right) }}\right] _n$ is also likely to be large. This observation implies that to reduce the peaks in $\vert \boldsymbol{f}_{\boldsymbol{b}^{\left( out\right) }} \vert _{\operatorname {mag}}$, $\vert \boldsymbol{f}_{\boldsymbol{b}^{\left( in\right) }} \vert _{\operatorname {mag}}$ should be as flat as possible.

To examine the influence of $\boldsymbol{x}_{v}$ subcarrier positions on the optimization results, we revisit the cost function presented in Eq. (22). This cost function comprises two terms: $\Vert \boldsymbol{W}_{t}^H \boldsymbol{b} \Vert _2$ and $\left( {\boldsymbol{y}_{v}^H\boldsymbol{b}+\boldsymbol{b}^H\boldsymbol{y}_{v}} \right) /{2}$. The second term promotes a large projection of the optimization variable $\boldsymbol{b}$ onto $\boldsymbol{y}_v$, which encourages the frequency-domain representation of $\boldsymbol{b}$ to match the preassigned values on the subcarriers indexed by $S_v$. At the same time, the first term encourages the spectral energy of $\boldsymbol{b}$ to be allocated on the subcarriers indexed by $S_t$. Therefore, maximizing the overall objective suggests that, in addition to matching the components on $S_v$, the spectral energy outside $S_v$ should be predominantly confined within $S_t$.

**Fig. 3:**
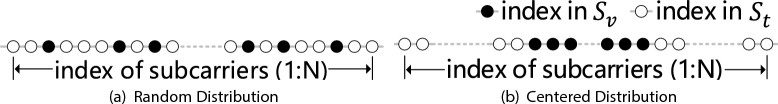
Distribution of ${\boldsymbol{x}}_{v}$ subcarrier positions.

Based on this structural observation, the relative positions of the subcarriers in $S_v$ and $S_t$ are not arbitrary. Two representative placement strategies for $S_v$ are therefore considered: distributing $S_v$ uniformly and randomly across the entire bandwidth, and placing $S_v$ at the center of the bandwidth with $S_t$ occupying the outer subcarriers. Both of strategies are illustrated in Fig. 3a and b respectively.

### The accelerated approaches

In the Basic Method, the iterative approach reduce $g_2\left( \boldsymbol{b}\right)$ by gradually guiding $\nabla g_2(\boldsymbol{\phi })$ to a zero vector. In successive iterations, ${\boldsymbol{b}}^{(k+1)}$ is updated by low-pass filtering ${\boldsymbol{b}}^{(k)}$, creating a certain correlation between them, which in turn leads to a correlation in the direction of changes in $\boldsymbol{\phi }$. As the iterative process converges to a local optimum, the changes in $\boldsymbol{\phi }$ between consecutive iterations become smaller, thereby strengthening this correlation. Using the correlation allows for predicting the next iteration result requiring only simple algebraic calculations.

**Fig. 4:**
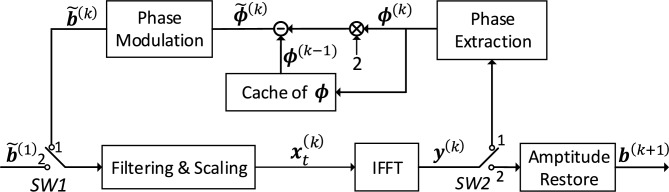
the block diagram of the Accelerated Method.

Figure 4 illustrates a scheme that exploits this correlation to improve algorithmic efficiency, referred to as the Accelerated Method. In the scheme, after updating $\boldsymbol{\phi }$ using the Basic Method, the updated values are stored in a buffer. When the next iteration occurs, the trend in $\boldsymbol{\phi }$ changes is determined by comparing with the previous results, allowing further updates to $\boldsymbol{\phi }$. Detailed steps of the Accelerated Method are provided in Algorithm 3. In this method, conversions between phase and complex variables can be efficiently implemented using CORDIC method.

**Algorithm 3:**
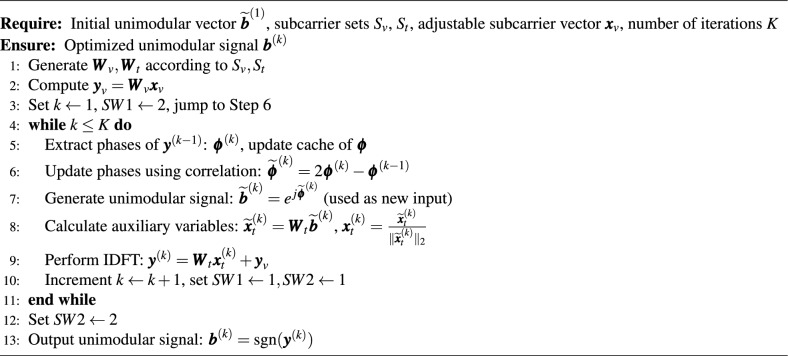
Accelerated PCA-L1 CVE Reduction Method

For each iteration, the primary computations include once FFT and once IFFT, of which computational complexity is $O_sN\log _{2}{\left( O_sN\right) }$. A lower oversampling rate $O_s$ reduces the computational load. However, if $O_s$ is too low, it may adversely affect the algorithm’s performance. In this study, the algorithm’s effectiveness is generally ensured when $O_s\ge 4$. $O_s$ is introduced to approximate the continuous-time envelope and does not alter the structure of the proposed optimization problem.4 it is related to the accuracy of envelope approximation rather than the proposed optimization framework itself. In practice, a sufficiently large oversampling rate is commonly adopted to ensure reliable envelope evaluation.

## Simulation and discussion

In this section, the proposed algorithm, denoted as $\textrm{TR} {-} \textrm{CVE}_{\textrm{rms}}$, is compared with several classical algorithms. The experiments aim to validate the superiority of the proposed method in envelope fluctuation suppression and power allocation control, as well as its insensitivity to initial conditions. The methods included in the comparison are as follows:

**Fig. 5:**
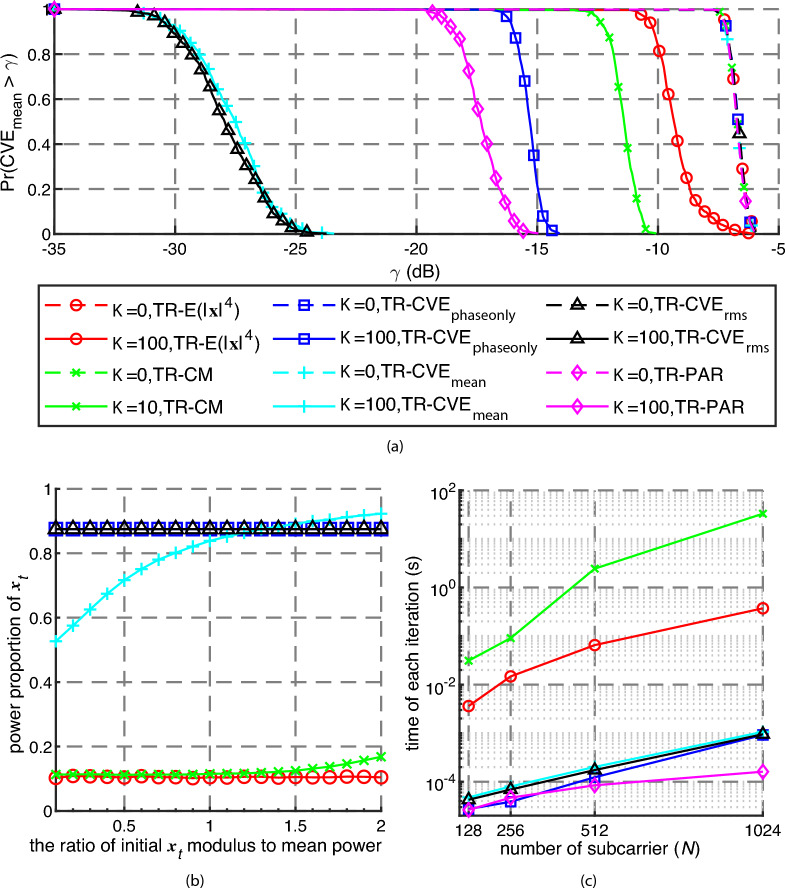
Comparisons between the proposed method and existing methods.

- $\textrm{TR} {-} \operatorname {E}\left( \vert x \vert ^ 4\right)$^30^: envelope fluctuations are suppressed by reducing the variation of instantaneous power and the number of iterations is set to 10.
- $\textrm{TR} {-} \textrm{CM}$^31^: an envelope fluctuations are suppressed by reducing the cubic metric, problem is resolved by Newton’s method and the number of iterations is set to 100.
- $\textrm{TR} {-} \textrm{CVE}_{\textrm{mean}}$^19^: the method reduces $\textrm{CVE}_{\textrm{mean}}$ by using a iterative least squares algorithm, the number of iterations is set to 100.
- $\textrm{TR} {-} \textrm{CVE}_{\textrm{phaseonly}}$^32^: the method improves the disadvantage of the uncontrolled PSD of $\textrm{TR} {-} \textrm{CVE}_{\textrm{mean}}$ method, the magnitude of each weight remains constant across iterations, with only the phase varying.
- $\textrm{TR} {-} \textrm{PAR}$ : This is a PAR reduction method based on clipping, with a clipping ratio of 0.2. It is selected as a representative classical approach for comparison with the other methods.

In all the simulations, $N=128, N_{v}=16, O_s=2$, the position of $x_{v}$ subcarrier is randomly selected. *K* is iteration rounds.

### Performance comparison

To evaluate the performance of the proposed method in suppressing envelope fluctuations, Fig. 5a presents the complementary cumulative distribution function (CCDF) of repeated experimental results obtained by different methods. The comparison metric is ${\textrm{CVE}}_{\textrm{mean}}$, which quantifies, in the least-squares sense, the proximity of a waveform to a constant-envelope signal. As shown in the figure, both $\textrm{TR}{-}\textrm{CVE}_{\textrm{rms}}$ and $\textrm{TR}{-}\textrm{CVE}_{\textrm{mean}}$ exhibit significantly better performance, with the vast majority of results falling below –25 dB. However, due to the absence of a power constraint in the $\textrm{TR}{-}\textrm{CVE}_{\textrm{mean}}$ algorithm, the resulting power distribution becomes inconsistent. As illustrated in Fig. 5b, the proportion of power allocated to the $x_{t}$ subcarriers varies with the choice of initialization point, which hinders uniform power distribution across subcarriers. Based on the analysis of Fig. 5a and b, $\textrm{TR}{-}\textrm{CVE}_{\textrm{rms}}$ is more suitable for applications requiring power uniformity.

**Fig. 6:**
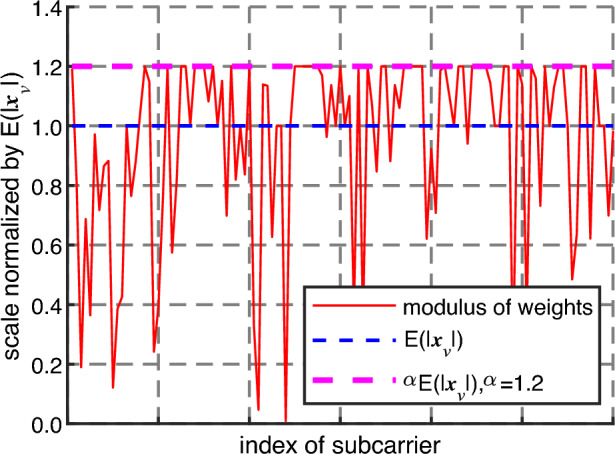
Magnitude of the weights obtained when $\alpha = 1.2$.

**Fig. 7:**
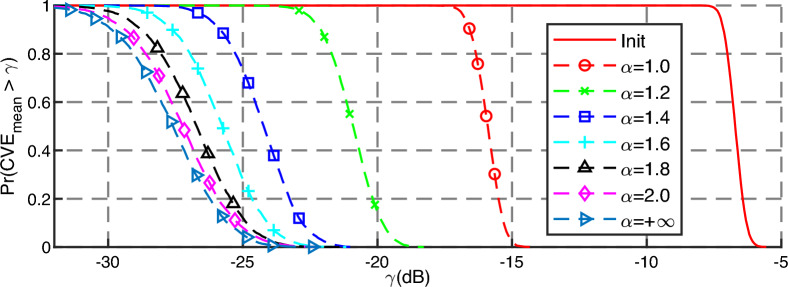
Performance of the Basic Method under different SPR constraints.

### Computational complexity

Figure 5c demonstrates the variation in the time required for a single iteration as a function of the number of subcarriers. It is important to note that the vertical axis is logarithmic. For the $\textrm{TR} {-} \textrm{CVE}_{\textrm{rms}}$ method, each iteration involves one pair of FFT/IFFT operations and once phase detection for a complex vector. The computational complexity is comparable to that of $\textrm{TR} {-} \textrm{CVE}_{\textrm{mean}}$ and $\textrm{TR} {-} \textrm{CVE}_{\textrm{phaseonly}}$ method. In contrast, the $\textrm{TR} {-} \operatorname {E}\left( \vert x \vert ^ 4\right)$ and $\textrm{TR} {-} \textrm{CM}$ methods require once matrix inversion in each iteration, the computational load is significantly increased.

### The impact of weight magnitude constraints

In section III, the weight magnitude constraints are involved, which may generally lead to a reduction in the performance of envelope fluctuation suppression. To illustrate this effect, an upper limit for magnitude is defined parametrically, $x_{max}=\alpha \operatorname {E}(\vert \boldsymbol{x}_{v} \vert )$. Figure 6 shows an example of the optimization result when $\alpha =1.2$.

Figure 7 further presents the distribution of CVE under optimization results for $\alpha =$ 1.0/1.2/1.4/1.6/1.8/2.0/+$\infty$. As $\alpha$ decreases, the optimization results progressively deteriorate, which aligns with intuitive expectations. A lower $\alpha$ generally corresponds to a flatter power spectrum, which enhances autocorrelation performance. In practice, the threshold can be flexibly adjusted according to requirements to achieve optimal overall performance.

### The impact of initial points selection

**Fig. 8:**
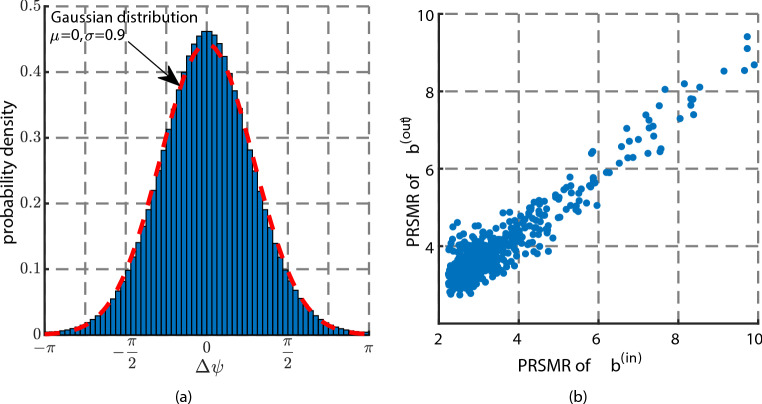
**a** Statistics of the $\Delta {\boldsymbol{\psi }}$ distribution; **b** The relationship between the peak magnitude in the spectra of ${\boldsymbol{b}}^{(in)}$ and $\boldsymbol{b}^{(out)}$.

In the discussion of initial point, $\Delta {\boldsymbol{\psi }}$ is assumed to follow a Gaussian distribution, which is confirmed through Monte Carlo simulations here. Figure 8a illustrates the statistical distribution of $\Delta \boldsymbol{\psi }$ obtained from 1000 repeated experiments. The dark curve in the figure represents the probability density function (PDF) of a normal distribution fitted using MATLAB, with a mean of 0 and a variance of approximately 0.9 (equivalent to $50^\circ$). The scatter plot presented in Figure 8b illustrates the relationship between the peak magnitudes in the spectra of ${\boldsymbol{b}}^{(in)}$ and $\boldsymbol{b}^{(out)}$. Specifically, each point in the plot corresponds to a single experiment, with the *x*- and *y*-coordinates representing the peak-to-RMS of spectral magnitude ratio (PRSMR) of the input and output unimodular signal, respectively, and PRSMR=$\frac{\operatorname {max}\vert \operatorname {DFT}\left( \boldsymbol{b}\right) \vert }{\operatorname {RMS}\left( \operatorname {DFT}\left( \boldsymbol{b}\right) \right) }$. The experimental results reveal a positive correlation between the peaks in the input and output spectrum, which aligns with the analysis in Section III.

**Fig. 9:**
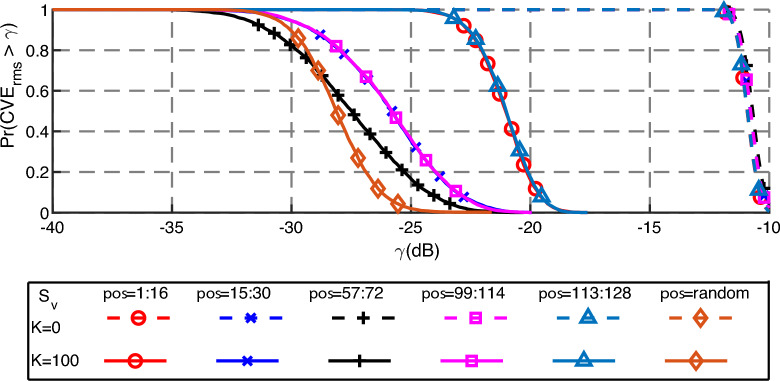
CCDF comparison under different $\boldsymbol{x}_{v}$ positions.

Figure 9 presents the CCDF of resulting $\operatorname {CVE}_{\textrm{rms}}$ for different $\boldsymbol{x}_{v}$ positions. The results indicate that the optimization performs best when the $\boldsymbol{x}_{v}$ subcarriers are either randomly distributed or centrally located.

**Fig. 10:**
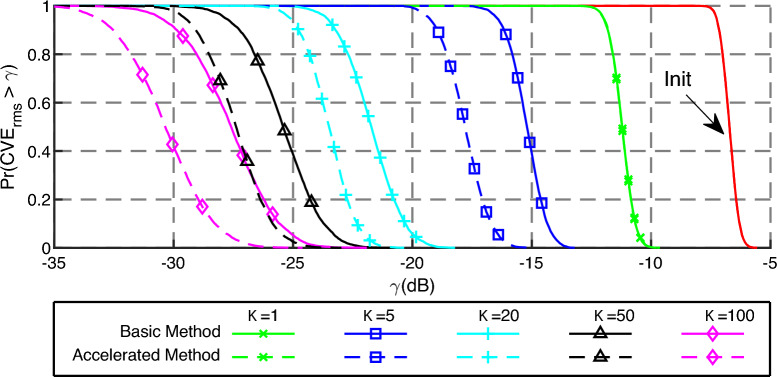
Comparisons between the Basic Method and the Accelerated Method.

### The performance of accelerated method

Figure 10 presents the CCDF of CVE obtained using the Basic Method and the Accelerated Method for different iteration counts *K*, where $K=$ 1, 5, 20, 50 and 100 respectively. The results highlight the superior performance of the Accelerated Method. At the initial stage $(K=1)$, the CCDF curves for both methods are nearly identical, indicating comparable performance. However, as the number of iterations increases, the accelerated method exhibits a significantly faster reduction in CVE. By $K=5$, the average CVE achieved by the Accelerated Method is approximately 3dB lower than that of the Basic Method. This means that the Accelerated Method can achieve the desired CVE reduction with fewer iterations, making it more efficient in practical applications.

## Conclusion

In this work, we present an OFDM waveform design mechanism that balances envelope optimization and subcarrier constraints. The improved metric $\operatorname {CVE}_{\textrm{rms}}$ is a important component of this study, which is particularly suitable for scenarios that require a constant envelope waveform. Compared to other metrics, $\operatorname {CVE}_{\textrm{rms}}$ can be further simplified in the presence of a total power constraint. In the proposed mechanism, the upper bounds of subcarrier weight magnitudes can be flexibly controlled via the parameter $x_{max}$, enabling adjustable uniformity in the subcarrier power distribution. Numerical results also reveal that achieving both a flat envelope and uniform power allocation often presents conflicting goals,which inherently limits the feasible optimization space. However, MIMO radars offer additional degrees of freedom. The incorporation of $\operatorname {CVE}_{\textrm{rms}}$ into MIMO radar waveform design will be explored in our future work.

## Data Availability

The data generated during this study are included in this article. Matlab code is available from the corresponding author on reasonable request.

## Appendix A: Proof of the sufficient condition

For a strict local maximum, the conditions require that $\nabla g_2 \left( \boldsymbol{\phi }\right) = \boldsymbol{0}$ and $\nabla ^2 g_2 \left( \boldsymbol{\phi }\right)$ is negative definite. When condition (31) is satisfied, the first-order condition is self-evident. To prove the second-order condition, denote the first and second parts of (30) as $H_1 (\boldsymbol{\phi })$ and $H_2 (\boldsymbol{\phi })$ respectively. For any $\Delta \boldsymbol{\phi }$, the quadratic form of the Hessian matrix can be expressed as $\Delta \boldsymbol{\phi }^T H_1 (\boldsymbol{\phi }) \Delta \boldsymbol{\phi }-\Delta \boldsymbol{\phi }^T H_2 (\boldsymbol{\phi }) \Delta \boldsymbol{\phi }$.

45 $$\begin{aligned} \begin{aligned}&\Delta \boldsymbol{\phi }^T H_1 (\boldsymbol{\phi }) \Delta \boldsymbol{\phi }\\ =&\frac{\Re \left\{ \left\| \sum _{n = 1}^{O_sN}{{{{\textbf {w}}}}_n b_n^* \Delta \phi _n} \right\| _2^2 - \sum _{n=1}^{O_sN}{\vert \Delta \phi _n \vert ^2 b_n^{*}\left( \left[ {\boldsymbol{W}}_{t} {\boldsymbol{W}}_{t}^H {\boldsymbol{b}}\right] _{n} + \left[ {\boldsymbol{y}}_{v} \right] _n \Vert {\boldsymbol{W}}_{t}^H \boldsymbol{b} \Vert _2 \right) } \right\} }{\left\| {\boldsymbol{W}}_{t}^{H} {\boldsymbol{b}} \right\| _2}\\ \le&\displaystyle \sum _{n=1}^{O_sN}{\frac{\Re \left\{ \vert \Delta \phi _n \vert ^2 \left[ \Vert {{\textbf {w}}}_n b_n^* \Vert _2^2 - b_n^{*}\left( \left[ {\boldsymbol{W}}_{t} {\boldsymbol{W}}_{t}^H {\boldsymbol{b}}\right] _{n} + \left[ {\boldsymbol{y}}_{v} \right] _n \Vert {\boldsymbol{W}}_{t}^H \boldsymbol{b} \Vert _2 \right) \right] \right\} }{\left\| {\boldsymbol{W}}_{t}^{H} {\boldsymbol{b}} \right\| _2}}\\ =&\frac{-1}{\left\| {\boldsymbol{W}}_{t}^{H} {\boldsymbol{b}} \right\| _2}\displaystyle \sum _{n=1}^{O_sN}{\Re \left\{ \vert \Delta \phi _n \vert ^2 \left[ b_n^{*} \left( \left[ {\boldsymbol{W}}_{t} {\boldsymbol{W}}_{t}^H {\boldsymbol{b}}\right] _{n} - \Vert {{{\textbf {w}}}}_n \Vert _2^2 b_n + \left[ {\boldsymbol{y}}_{v} \right] _n \Vert {\boldsymbol{W}}_{t}^H \boldsymbol{b} \Vert _2 \right) \right] \right\} } \end{aligned} \end{aligned}$$

Substituting condition (31), we can deduce that $\Delta \boldsymbol{\phi }^T H_1 (\boldsymbol{\phi }) \Delta \boldsymbol{\phi }< 0$. Similarly, the quadratic form of $H_2 (\boldsymbol{\phi })$ is,

46 $$\begin{aligned} \begin{aligned}&\Delta \boldsymbol{\phi }^T H_2 (\boldsymbol{\phi }) \Delta \boldsymbol{\phi }\\ =&\Delta \boldsymbol{\phi }^T \frac{\Im \left\{ {\boldsymbol{b}}^{*} \odot \left[ {\boldsymbol{W}}_{t} {\boldsymbol{W}}_{t}^H \boldsymbol{b} \right] \right\} \left( \Im \left\{ {\boldsymbol{b}}^{*} \odot \left[ {\boldsymbol{W}}_{t} {\boldsymbol{W}}_{t}^H \boldsymbol{b} \right] \right\} \right) ^T}{\Vert {\boldsymbol{W}}_{t}^H \boldsymbol{b} \Vert _2^3} \Delta \boldsymbol{\phi }\\ \end{aligned} \end{aligned}$$

Obviously, $\Delta \boldsymbol{\phi }^T H_2 (\boldsymbol{\phi }) \Delta \boldsymbol{\phi }\ge 0$. Therefore, $\Delta \boldsymbol{\phi }^T \left[ H_1 (\boldsymbol{\phi })-H_2 (\boldsymbol{\phi }) \right] \Delta \boldsymbol{\phi }< 0$ when condition (31) is satisfied, which indicates $\nabla ^2 g_2 \left( \boldsymbol{\phi }\right)$ is negative definite and the corresponding $\boldsymbol{b}$ is a local maximum.
